# Preliminary toxicity results using partial breast 3D-CRT with once daily hypo-fractionation and deep inspiratory breath hold

**DOI:** 10.1186/s13014-018-1079-x

**Published:** 2018-07-27

**Authors:** Roman O. Kowalchuk, Kara D. Romano, Daniel M. Trifiletti, Sunil W. Dutta, Timothy N. Showalter, Monica M. Morris

**Affiliations:** 1 Department of Radiation Oncology, University of Virginia, 1240 Lee Street, Box 800383, Charlottesville, VA 22908 USA; 2 Department of Radiation Oncology, Virginia Commonwealth University, Richmond, VA USA

**Keywords:** Breast cancer, Radiotherapy, Hypo-fractionation, Acute toxicity

## Abstract

**Background:**

To evaluate the clinical outcomes of patients treated with 3D conformal **H**ypo-fractionated, deep **I**nspiratory breath-hold (DIBH), **P**artial breast radiotherapy, termed “HIP.” HIP was implemented to merge the schedule of once-daily breast hypofractionation with partial breast treatment.

**Methods:**

We identified 38 breast cancers in 37 patients from 2013 to 2014 treated at our institution with HIP following lumpectomy for early stage breast cancer. Patients received a hypo-fractionated course (≤ 20 fractions) of once daily radiation to the partial breast (lumpectomy cavity + margin) utilizing DIBH regardless of laterality. Clinical and treatment-related characteristics were obtained, including target volume and organ at risk (OAR) dosimetric characteristics. Patients were followed clinically and with at least yearly mammograms for up to 36 months (range 5–36 months). Acute and late toxicity was scored using the Common Terminology Criteria for Adverse Events (CTCAE) v4.03.

**Results:**

Patients received a median dose of 42.56 Gy in 16 Fractions (Fx) (range 40.05–53.2 Gy; and 15–20 Fx). OAR doses were low, with a mean heart dose of 0.37 Gy, an ipsilateral lung V20 mean of 4%, and a contralateral lung V5 of 1%. Acute toxicity (≤ grade 2) was present in 79% (*n* = 30) of the cases, with dermatitis being the most common finding (63%). Late grade 1–2 toxicity was present in 42% (*n* = 16) of the cases, with hyperpigmentation being the most common finding (*n* = 9). There were no severe acute or late toxicities (≥ grade 3). At a median follow up of 21 months, there were no local, regional, or distant failures.

**Conclusions:**

We report limited toxicity in this low risk cohort of patients with early stage breast cancer treated with HIP, a unique and logical combination of 3-D conformal external beam radiotherapy, moderate hypo-fractionation, and DIBH.

## Background

Breast cancer is the most commonly diagnosed malignancy among women in the United States. In 2013, there were an estimated 232,340 new cases accounting for 29% of all newly diagnosed cancers [1]. Breast conservation therapy (BCT), which includes lumpectomy (surgical resection of the tumor alone) and radiation therapy (RT), is the nationally accepted standard treatment approach for early stage breast cancer and is used in 70% of such patients [2, 3].

The use of adjuvant whole breast irradiation (WBI) following breast conservation surgery has been demonstrated in numerous clinical trials to reduce the rates of ipsilateral breast tumor recurrence (IBTR) by over 50% compared to lumpectomy alone [4, 5]. The advantage of BCT over mastectomy is avoidance of a larger surgical procedure, shorter surgical recovery time, superior cosmetic outcomes, and organ preservation; however, the disadvantage of adjuvant WBI is the prolonged treatment time of 3–7 weeks and additional radiation-induced toxicities. With strong clinical evidence to support its use, hypo-fractionation has become increasingly popular for patients in Canada and the United States for early stage breast cancer [2, 6, 7]. There is sufficient evidence from randomized clinical trials that hypo-fractionated RT is now considered the national preferred option for the majority of patients [6, 8, 9].

Approximately 75% of breast tumor recurrences occur within or near the lumpectomy cavity, triggering attempts to decrease the volume of breast treated with radiation [10–12]. PBI in conjunction with further hypofractionation, termed accelerated partial breast irradiation (APBI) can be delivered in several forms – brachytherapy with multicatheter balloons, brachytherapy with interstitial implants, or 3-dimensional conformal external beam radiotherapy (3D-CRT). Early data suggests that APBI is safe and effective, and it has been endorsed by the NCCN Guidelines [2]. Various selection criteria exist to guide clinicians in choosing the optimal patients for this technique, but generally, women at sufficient risk for local failure warranting adjuvant therapy, but not regional or distant failure, should be considered [13–16].

The optimal APBI dose and fractionation is currently unclear. The National Surgical Adjuvant Breast and Bowel Project (NSABP) B-39 clinical trial used a dose of 38.5 Gy in 10 fractions delivered twice daily for 3D-CRT and 34 Gy in 10 fractions for brachytherapy [17]. The 38.5 Gy dose was chosen as the biologically equivalent dose (BED) to 45 Gy in 25 fractions assuming an alpha/beta ratio of 10. This same dose was used in the Radiation Therapy Oncology Group (RTOG) 0319 clinical trial [18]. However, this dose and fractionation has led to concerns regarding the increased incidence of late cosmetic toxicity, including subcutaneous fibrosis and fat necrosis [19]. Further, some patients and providers may prefer once-daily fractionation schedules over twice-daily. IMRT has also been used with APBI, notably in the Florence NCT02104895 trial. A dose of 30 Gy in 5 daily fractions was compared to WBI of 50 Gy in 25 fractions, with a boost of 10 Gy in five fractions. In 520 patients, no significant difference was found in ipsilateral breast tumor recurrence or overall survival, and improved acute and late cosmetic outcomes were found in the APBI group [20]. The use of interstitial multicatheter brachytherapy for APBI has been studied, and Strnad et al. presented five year results of non-inferiority of local recurrence (1.44%) and side effects [21].

Taken together, there is strong evidence for the use of moderate hypo-fractionation (15–20 fractions) and PBI. A technique for hypo-fractionated PBI using 3D-CRT with deep inspiratory breath hold (HIP: hypo-fractionated inspiratory partial breast irradiation) was implemented at our institution. The 3D-CRT PBI is a particularly useful treatment technique, as it is applicable and readily available to nearly all centers. HIP merged two types of radiation therapy in a novel way by offering “Canadian” hypo-fractionation (15–20 daily fractions) with a partial breast volume. Deep inspiration breath-hold (DIBH) was employed to reduce cardiac dose [22–24] and improve target volume coverage by immobilizing the breast and decreasing toxicity to the ipsilateral lung [25].

This study seeks to identify early clinical outcomes of early stage breast cancer, including efficacy and toxicity associated with HIP.

## Methods

### Data collection

We conducted a retrospective analysis of 37 patients with 38 breast cancers treated with3D-CRT, hypo-fractionated PBI from 2013 to 2014 under our uniform institutional policy for HIP during this time period. Approval was obtained from institutional review board (IRB) prior to evaluating outcomes for these patients.

Clinical data was recorded for all eligible patients including: age, stage, histology, grade, estrogen/progesterone receptor status, Her-2-neu amplification status, lympho-vascular space invasion, multifocal disease, surgical margins, menopausal status, and adjuvant therapies (endocrine therapy or chemotherapy).

### Treatment planning

#### CT simulation and technique

All patients underwent a computed tomography (CT) simulation positioned on a breast board in the supine position with arms raised overhead and a Vac-Lok (Med Tech Inc., Orange City, IA) for custom immobilization. Radio-dense markers were placed at the time of simulation to delineate the visible borders of breast tissue and the lumpectomy scar. DIBH technique was used for all patients regardless of laterality to increase the accuracy of the target volume location and reduce heart dose, as previously reported [26]. Patients were positioned with both arms raised over the head and custom immobilization. Then, the Varian real-time position management (RPM) system was used to initiate DIBH imaging and monitor the duration and displacement of each patient’s breath hold. This RPM signal was used as a baseline, and upper and lower limits of 0.5 cm from the baseline were established, such that treatment would only take place if the patient’s breath hold was within this displacement gate. Free breathing was allowed between treatment fields or if multiple breath holds were required for a given field [26]. All patients were treated with PBI using a once-daily fractionation schedule with a 3D conformal plan using multiple non-coplanar beams. Image-guidance included first day MV portal imaging and daily kV imaging to verify positioning and alignment.

#### Target volumes

**Fig. 1:**
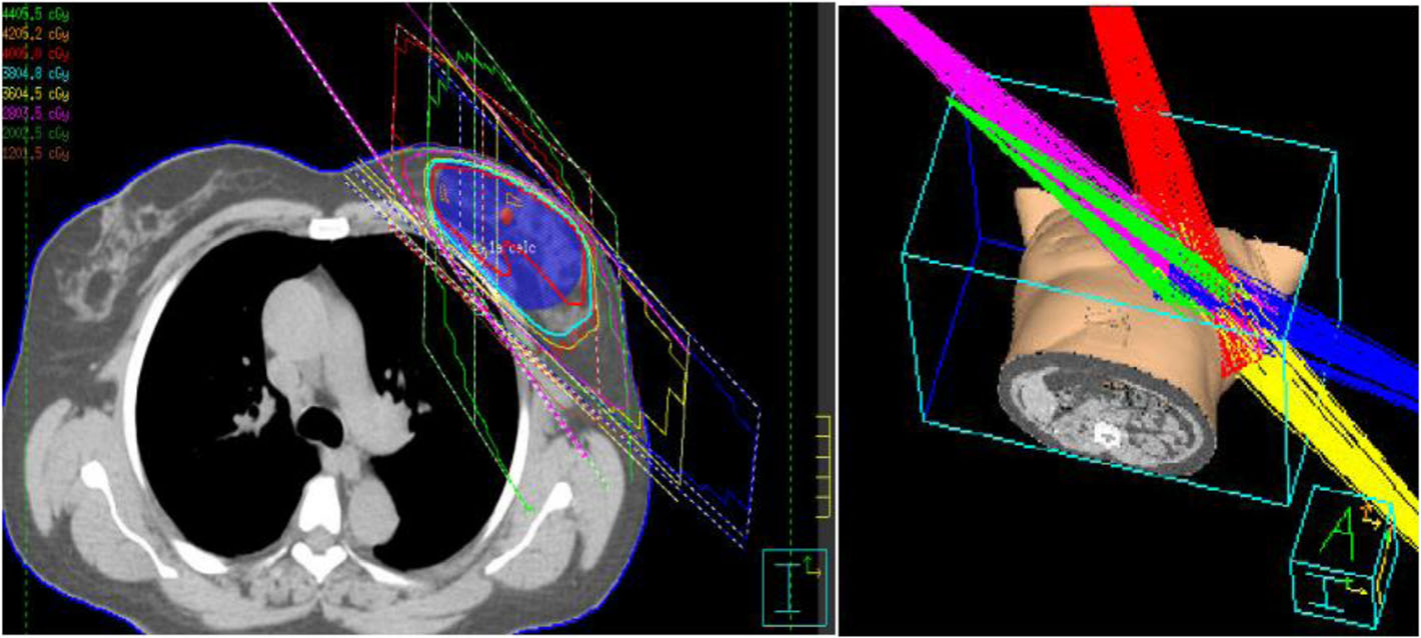
Here is an example of an HIP plan utilizing 5 non-coplanar 3D beams to create a conformal dose distribution to the partial breast to a total dose of 40.05 Gy in 15 Fx

The partial breast target volumes were based on the RTOG 1005 and NSABP B-39 clinical trials [17, 27]. The lumpectomy gross tumor volume (GTV) included all clinical and radiographic information of the excision cavity, architectural distortion, lumpectomy scar, seroma, and/or surgical clips. The use of surgical clips is standard for such cases at this institution. The lumpectomy clinical target volume (CTV) was defined as GTV + 10–15 mm uniform expansion and this was limited posteriorly at the anterior surface of the pectoralis muscles and anteriorly 5 mm from skin. The lumpectomy planning target volume (PTV) was defined as CTV + 7 - 10 mm. The beam apertures were chosen based on the PTV. To evaluate our target volume coverage and dose-volume constraints, the PTV was copied to create a PTV_Eval. The PTV_Eval was defined as PTV excluding the region outside of breast tissue, i.e. cropped posteriorly at the anterior border of the pectoralis muscles and anteriorly 5 mm from skin. One characteristic treatment plan is shown in Fig. 1.

#### Plan evaluation

Treatment related characteristics and dosimetric data were recorded including: laterality, total dose, number of fractions, and organ at risk data including heart mean dose, volume of heart receiving 20 Gy or more (heart V20), volume of ipsilateral lung receiving 20 Gy or more (ipsilateral lung V20), volume of contralateral lung receiving 5 Gy or more (contralateral lung V5), contralateral breast maximum and mean.

### Treatment outcomes

During treatment, patients were evaluated by a physician at least once weekly, including the radiation and breast oncology teams. Clinical follow up data includes history, physical exam, and mammograms. Breast cancer recurrence was recorded as: ipsilateral breast tumor recurrence (IBTR) (in the PTV, outside the PTV, or skin), ipsilateral nodal recurrence, contralateral breast failure, or distant failure.

Toxicity was graded according to the Common Toxicity Criteria for Adverse Events (CTCAE v4.03) to evaluate both acute (≤ 90 days post-treatment) and late (> 90 days post-treatment) findings [28]. Toxicities were reported as fatigue, breast pain, dermatitis, pruritus, skin hyper/hypo-pigmentation, skin induration, skin ulceration, telangiectasia, breast fibrosis, lung complications, heart complications, and lymphopenia.

## Results

### Clinical characteristics

Of the 38 breast cancers in this study, 23.7% were stage 0, 86.8% were stage IA, 0% were stage IB, and 7.9% were stage IIA. Patient and tumor characteristics are listed in Table 1. All patients were deemed “suitable” or “cautionary” per ASTRO consensus guidelines for APBI [16]. While 78.9% of the patients were considered cautionary per the 2009 recommendations, this number decreased to 36.8% in the updated 2016 recommendations, as seen in Table 2 [29]. The recent update stated that ductal carcinoma in situ (DCIS) is suitable for APBI if it is screening-detected, low to intermediate nuclear grade, size ≤2.5 cm, and resected with margins negative at ≥3 mm. Of the cautionary patients, 43% were estrogen receptor negative, and 36% had invasive lobular carcinoma (ILC) on histologic review. Clinical outcomes using APBI with ILC have been found to be no different than those with invasive ductal histology, hence their inclusion in this study [30]. There were no unsuitable patients treated in this study.

**Table 1:** Patient and tumor characteristics of 38 breast cancers and 37 patients

Characteristics	n	%
Female	38	100
Age (median, years)		62 years (range: 52–79)
Laterality		
Right	18	47
Left	20	53
Menopausal status		
Pre-menopausal	0	
Post-menopausal	37	97
Unknown	1	3
Surgical margin		
Negative	37	97
Positive	1	3
Surgery, breast		
Lumpectomy	38	100
Mastectomy	0	
Surgery, Axilla		
None	11	29
SLNBx	27	71
ALND	0	
Histology		
DCIS	12	32
Invasive Ductal Carcinoma	21	55
Invasive Lobular Carcinoma	5	13
T stage		
Tis	9	24
T1a	5	13
T1b	9	24
T1c	12	32
T2	3	8
N stage		
N0	38	100
M stage		
M0	38	100
LVSI		
No	11	29
Yes	0	
Not reported	27	71
Grade		
1	8	21
2	21	55
3	9	24
ER+	32	84
Unknown	0	
PR+	20	53
Unknown	8	21
HER2+	2	5
Unknown	12	32
Multi-focal	14	37
Hormonal therapy	28	74
Chemotherapy	5	13

*Abbreviations*: *SLNBx* sentinel lymph node biopsy, *ALND* axillary lymph node dissection, *DCIS* ductal carcinoma in situ, *LVSI* lympho-vascular space invasion, *ER* estrogen receptor, *PR* progesterone receptor, *HER2* human epidermal growth factor receptor 2

**Table 2:** Patient category according to ASTRO consensus guidelines for APBI

ASTRO consensus	Category	n	%
APBI Group (2009 Original)	Suitable	8	21.1
	Cautionary	30	78.9
	Unsuitable	0	
APBI Group (2016 Update)	Suitable	24	63.2
	Cautionary	14	36.8
	Unsuitable	0	

*Abbreviations*: *ASTRO* American Society for Radiation Oncology

### Treatment characteristics

The median total dose was 42.56 Gy (range of 40.05–53.20 Gy), over a median 16 Fx (range of 15–20 Fx). Table 3 provides radiation treatment characteristics of the entire cohort.

**Table 3:** Radiation treatment characteristics of 38 breast cancers treated with HIP

Characteristics	Value
Total Dose (mean, Gy)	43.54 (range: 40.05–53.20)
Total Dose (median, Gy)	42.56
Total Fractions (mean, Fx)	16 (range: 15–20)
Total Fractions (median, Fx)	16
Modality	
Photon only	33 (86.8%)
Mixed beam (photon/electron)	5 (13.2%)
Heart mean dose (median, Gy)	0.37 (range: 0.14–0.94)
Heart mean dose, right breast (mean, Gy)	0.36
Heart mean dose, left breast (mean, Gy)	0.38
Heart V20 (mean, %)	0
Heart V20 (median, %)	0
Ipsilateral lung V20 (median, %)	4.0
Contralateral lung V5 (median, %, *n* = 17)	1.3
Contralateral breast Dmax (median, Gy, *n* = 13)	1.4
Contralateral breast mean (median, Gy, *n* = 13)	0.2

*Abbreviations*: *Dmax* maximum point dose

### Toxicity

Toxicity data is listed in Table 4. Acute toxicity of any kind was recorded in 30 of 38 cases (79%) within 90 days of completion of treatment. There were no grade 3 or greater acute toxicities. Overall, acute dermatitis (58% grade 1 and 5% grade2) was the most common finding, followed by fatigue (8% grade 1 and 5% grade 2) and hyperpigmentation (13% grade 1). There were no cases of hypopigmentation, ulceration, telangiectasia, or fibrosis.

**Table 4:** Toxicity following 3D-CRT external beam HIP (according to CTCAE v 4.0)

	Grade 1	Grade 2	Grade 3–5
Acute Toxicity^a^ (*n* = 30 cases)			
Dermatitis	22	2	0
Pruritus	1	0	0
Hyperpigmentation	5	0	0
Hypopigmentation	0	0	0
Induration	2	0	0
Ulceration	0	0	0
Telangiectasia	0	0	0
Fibrosis	0	0	0
Fatigue	3	2	0
Pain	4	1	0
Late Toxicity^b^ (*n* = 16 cases)			
Dermatitis	0	0	0
Pruritus	0	0	0
Hyperpigmentation	0	0	0
Hypopigmentation	9	0	0
Induration	1	0	0
Ulceration	0	0	0
Telangiectasia	0	0	0
Fibrosis	8	0	0
Fatigue	1	0	0
Pain	0	0	0

^a^≤ 90 days from the start of radiation therapy

^b^> 90 days from the start of radiation therapy

Late toxicity of any kind was recorded in 16 of 36 cases (42%). Long term data was not available for 2 cases. There were no grade 2 or greater late toxicities. Only grade 1 toxicity was noted, and it was most commonly hyperpigmentation (24%), followed by fibrosis (21%). There were no cases of late dermatitis, pruritus, hypopigmentation, ulceration, telangiectasia, or pain.

### Disease control

At a median follow up of 21 months, there were no local, regional, or distant failures, including IBTR in and out of the field, skin recurrence, and contralateral breast failure.

## Discussion

Our results demonstrate a favorable rate of acute and late toxicity following HIP. The Whelan Trial reported comparable rates of good or excellent cosmetic outcomes in the control WBI group compared to the hypo-fractionated arm (71% vs. 70%) at 10 years of follow up [8]. The Royal Marsden Hospital/Gloucestershire Oncology Center (RMH/GOC) trial similarly found that 30.3–45.7% of patients recorded some change in breast appearance at 5 years [31]. Similarly, our results showed that 10 of 38 treatments resulted in breast appearance changes (26.3%). Further, our data showed no cases of telangiectasia, whereas the RMH/GOC trial showed rates of 13.8–14.3%.

Some concerns remain regarding late toxicity and poor cosmesis. The RAPID trial compared standard WBI to 38.5 Gy in 10 Fx external beam APBI, showing worse grade 1–2 toxicity and cosmesis in the experimental APBI arm at 3 years [32]. Conversely, the UK IMPORT LOW trial utilized a dose of 40 Gy in 15 Fx to the partial breast, and this trial not only found PBI at this dose to be non-inferior regarding local relapse but also adverse effects were similar to better when compared to WBI [33]. The PBI group had better late outcomes at 5 years regarding skin change, overall breast appearance, smaller breast, and breast firmness. Thus, although some data raises concern regarding APBI toxicity (e.g. the RAPID trial), there is a growing body of literature demonstrating non-inferior – or even superior – toxicity outcomes [33].

**Fig. 2:**
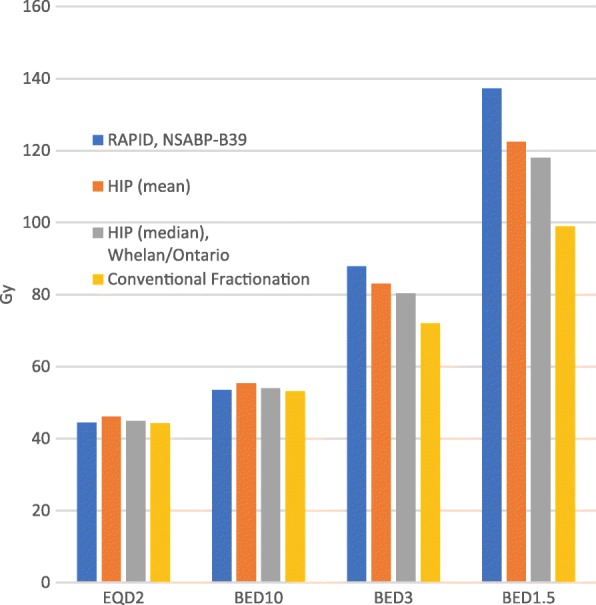
Dose comparisons of various dose/fractionation schedules for breast irradiation. Blue: 38.5 Gy in 10 Fx (RAPID and NSABP B-39 trials); Red: 43.54 Gy in 16 Fx(mean HIP dose); Green: 42.56 Gy in 16 Fx (median HIP dose, Whelan/Ontario hypo-fractionation); Purple: 45 Gy in 25 Fx (conventional fractionation). Abbreviations: EQD2: equivalent dose in 2 Gy fractions, BED_10_: biologically effective dose for tissue/tumor with an α/β ratio of 10, 3 and 1.5 respectively

The low incidence of late adverse effects reported in our study coupled with the improved cosmesis in the IMPORT LOW study may reflect the inherent differences of dose fractionation. Late-responding normal tissues (e.g. dermal skin) are characterized by a low α/β ratio. Figure 2 demonstrates that the frequently utilized dose fractionation schedules for PBI (including 38.5 Gy in 10 Fx) have similar EQD2 and BED10 values compared to standard WBI hypo-fractionation doses (used in both these patients and the IMPORT LOW study); however, the BED3 and BED 1.5 of normal tissues is substantially higher with the 38.5 Gy in 10 Fx schedule.

DIBH was developed to reduce cardiac dose. Epidemiological studies have found higher mortality from myocardial infarction with left-sided breast RT compared to right-sided, and reduction of cardiac dose has been shown to decrease ischemic heart disease [22–24]. In this study, heart V20 was 0%, and the mean heart dose was 0.37 Gy (range 0.14–0.94 Gy). Compared to brachytherapy APBI, DIBH WBI has been shown to have a lower heart dose [34]. In our study, the low heart dose is likely a combination of PBI and DIBH. DIBH also further immobilizes the breast during treatment, improving the accuracy of dose delivery and reducing radiation doses to the ipsilateral lung [35]. In this case, ipsilateral lung V20 had a mean of 4.0% (range 0–13).

The limitations of this study include the retrospective analysis by nature, which obfuscates detailed cosmetic analysis at long term intervals. The follow up of 22 months is likely insufficient time to capture many instances of IBTR, but it is sufficient to note acute toxicity and most late toxicity associated with RT. The cohort size is small, largely due to the unique scheduling and delivery method applied to this patient set.

## Conclusion

We report limited toxicity without early recurrences in this low risk cohort of patients with early stage breast cancer treated with HIP, a unique combination of 3D-CRT external beam radiotherapy, moderate hypo-fractionation, and DIBH. HIP offers early recurrence and toxicity findings consistent with previous PBI trials, such as IMPORT LOW, and the addition of DIBH offers the potential to further reduce patient motion and toxicity.

## Abbreviations

ALND: Axillary lymph node dissection
APBI: Accelerated partial breast irradiation
ASTRO: American Society for Radiation Oncology
BCT: Breast conservation therapy
BED: Biologically equivalent dose
BED_10_: Biologically effective dose for tissue/tumor with an α/β ratio of 10, 3 and 1.5, respectively
CT: Computed tomography
CTCAE v4.03: Common Toxicity Criteria for Adverse Events
CTV: Clinical target volume
DCIS: Ductal carcinoma in situ
DIBH: Deep inspiratory breath-hold
Dmax: Maximum point dose
EQD2: Equivalent dose in 2 Gy fractions
ER: Estrogen receptor
Fx: Fractions
GTV: Gross tumor volume
Gy: Gray
HER2: Human epidermal growth factor receptor 2
HIP: **H**ypo-fractionated, deep **I**nspiratory breath-hold, **P**artial breast radiotherapy
IBTR: Ipsilateral breast tumor recurrence
IRB: Institutional review board
LVSI: Lympho-vascular space invasion
NSABP: National Surgical Adjuvant Breast and Bowel Project
PBI: Partial breast irradiation
PR: Progesterone receptor
PTV: Planning target volume
RMH/GOC: Royal Marsden Hospital / Gloucestershire Oncology Center
RT: Radiation therapy
RTOG: Radiation Therapy Oncology Group
SLNBx: Sentinel lymph node biopsy
V20: Volume of an organ receiving at least 20 Gy
V5: Volume of an organ receiving at least 5 Gy
WBI: Whole breast irradiation
